# New Insights Into CRASP-Mediated Complement Evasion in the Lyme Disease Enzootic Cycle

**DOI:** 10.3389/fcimb.2020.00001

**Published:** 2020-01-30

**Authors:** Yi-Pin Lin, Amber M. Frye, Tristan A. Nowak, Peter Kraiczy

**Affiliations:** ^1^Department of Biomedical Sciences, State University of New York at Albany, Albany, NY, United States; ^2^Division of Infectious Diseases, New York State Department of Health, Wadsworth Center, Albany, NY, United States; ^3^Institute of Medical Microbiology and Infection Control, University Hospital, Goethe University Frankfurt, Frankfurt, Germany

**Keywords:** *Borrelia*, complement, Factor H, CspA, CspZ, OspE, tick, host-pathogen interaction

## Abstract

Lyme disease (LD), which is caused by genospecies of the *Borrelia burgdorferi* sensu lato complex, is the most common vector-borne disease in the Northern hemisphere. Spirochetes are transmitted by *Ixodes* ticks and maintained in diverse vertebrate animal hosts. Following tick bite, spirochetes initially establish a localized infection in the skin. However, they may also disseminate hematogenously to several distal sites, including heart, joints, or the CNS. Because they need to survive in diverse microenvironments, from tick vector to mammalian hosts, spirochetes have developed multiple strategies to combat the numerous host defense mechanisms. One of these strategies includes the production of a number of complement-regulator acquiring surface proteins (CRASPs) which encompass CspA, CspZ, and OspE paralogs to blunt the complement pathway. These proteins are capable of preventing complement activation on the spirochete surface by binding to complement regulator Factor H. The genes encoding these CRASPs differ in their expression patterns during the tick-to-host infection cycle, implying that these proteins may exhibit different functions during infection. This review summarizes the recent published reports which investigated the roles that each of these molecules plays in conferring tick-borne transmission and dissemination in vertebrate hosts. These findings offer novel mechanistic insights into LD pathobiology and may facilitate the identification of new targets for preventive strategies against Lyme borreliosis.

## Lyme Disease Spirochetes Evade the Vertebrate Hosts' Complement

**Figure 1:**
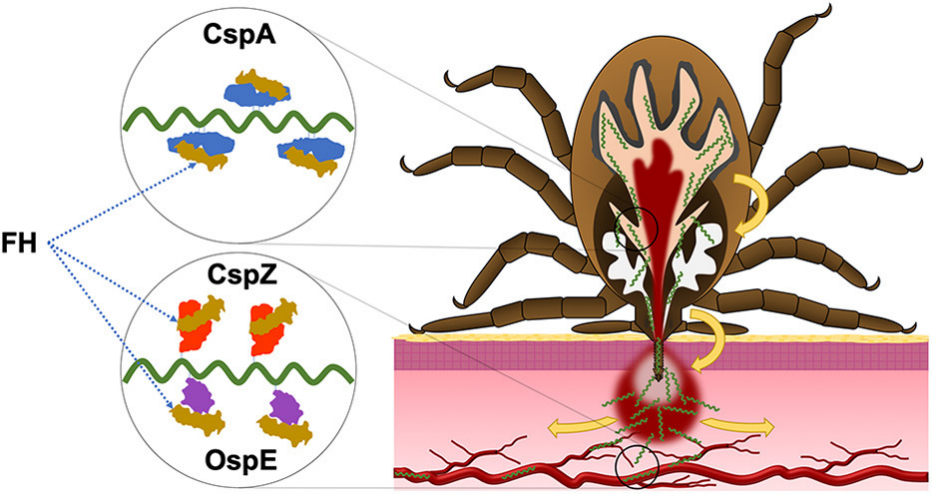
The roles of CRASP proteins in the enzootic cycle of LD spirochetes. During the infection, LD spirochetes require the ability to evade the complement in the vertebrate blood. CspA facilitates spirochete survival in the blood meal of fed ticks and thereby enabling spirochetes to be transmitted to the host. CspZ promotes spirochete survival in the bloodstream of vertebrate animals, allowing in dissemination to distal tissues. While the role that OspE paralogs (OspE) play in enzootic cycle remain unclear, the current evidence supports that these proteins confer spirochete dissemination in the vertebrate animals.

Lyme disease (LD) is the most common vector-borne disease in the northern hemisphere (Steere et al., 2016). A recent report from the CDC categorizes LD as one of the zoonotic diseases of the greatest concern in the United States. The disease is caused by spirochetes of the *Borrelia burgdorferi* sensu lato complex (Rosa et al., 2005; Brisson et al., 2012; Radolf et al., 2012). Among the ~20 *Borrelia* species that comprise the sensu lato complex, at least six have been confirmed to cause LD in humans including *Borrelia (B.) burgdorferi* sensu stricto (hereafter referred as *B. burgdorferi*)*, B. afzelii, B. garinii, B. spielmanii, B. bavariensis*, and *B. mayonii*, all of which are transmitted by *Ixodes* ticks and maintained in diverse reservoir hosts (mainly small mammals and birds) (Tufts et al., 2019). Upon tick feeding, spirochetes are exposed to host blood and the first line of innate immunity which they must overcome to survive (Hovius et al., 2007; Steere et al., 2016; Figure 1). Spirochetes then migrate through the tick midgut epithelium and the salivary glands and are transmitted to the host skin to establish the infection (Hovius et al., 2007; Steere et al., 2016; Figure 1). In untreated humans, the spirochetes may disseminate hematogenously to distal tissues and organs (Coburn et al., 2013; Hyde, 2017; Bernard et al., 2019; Figure 1).

Complement is a central component of the host innate immune system and the first line of defense against bacterial infection. Evasion of the host complement system is essential for *Borrelia* to successfully establish infection (Caine and Coburn, 2016; Kraiczy, 2016; Marcinkiewicz et al., 2017) (see Sjoberg et al., 2009; Zipfel and Skerka, 2009; Meri, 2016 for more thorough reviews). The complement system is composed of more than 30 proteins and inactive precursors (Zipfel and Skerka, 2009). Activation of complement cascades on the microbial surface is initiated via three distinct pathways (Meri, 2016). Antibody-antigen complexes trigger activation of the classical pathway (CP) whereas the mannose-binding lectin pathway (LP) is activated by recognition of carbohydrate complexes (collectins and ficolins) on microbial surfaces. The alternative pathway (AP) is activated when C3b is bound to the surface of invading microbes. Activation of all three pathways leads to the formation and deposition of C3 and C5 convertases on the microbial surface. This results in the insertion of the pore-forming membrane attack complex (MAC), leading to bacterial cell lysis.

In the absence of invading microbes or cell/tissue damage, vertebrate hosts produce complement regulatory proteins (CRPs) which are deposited on host cells/tissues to avoid non-specific damage by the complement cascade (Sjoberg et al., 2009; Zipfel and Skerka, 2009; Meri, 2016). Factor H (FH) is a CRP that binds to C3b by recruiting the serum protease, factor I. This complex leads to the degradation of C3b and coincidently terminates activation of AP (Zipfel and Skerka, 2009; Zipfel et al., 2013).

LD spirochetes produce several outer surface proteins that facilitate host complement evasion (de Taeye et al., 2013; Caine and Coburn, 2016; Kraiczy, 2016; Marcinkiewicz et al., 2017). These proteins include five complement-regulator acquiring surface proteins (BbCRASPs or CRASPs) (Kraiczy and Stevenson, 2013): CspA (CRASP-1, BBA68), CspZ (CRASP-2, BBH06), and OspE paralogs [i.e., ErpP (CRASP-3, BBN38), ErpC (CRASP-4), and ErpA/I/N (CRASP-5, BBP38, BBL39)] (Table 1). While all these proteins bind to FH to inactivate human complement, CspA and CspZ also bind to FH-like protein 1 (FHL-1), the truncated form of FH (Zipfel and Skerka, 1999; Kraiczy and Stevenson, 2013). Additionally, ErpP, ErpC, and ErpA bind to different FH-related proteins (CFHR), a family of CRPs with similar sequence identity and high-resolution structures to that of FH (Zipfel et al., 2002; Kraiczy and Stevenson, 2013). The expression of the genes encoding these outer surface proteins varies at different stages of the infection cycle, e.g., during spirochete transmission and dissemination (Miller et al., 2003; von Lackum et al., 2005; Bykowski et al., 2007; Brissette et al., 2008). These findings suggest that CRASPs play distinct roles in facilitating spirochete survival in ticks and/or vertebrate hosts. However, until recently, the role of these CRASPs in the spirochete infection cycle in vertebrate hosts is still unclear.

**Table 1:** *In vitro* and *in vivo* characteristics of CRASPs^a^^,^^b^.

		**CspA**	**CspZ**	**OspE paralogs**		
Synonyms and other designations		CRASP-1 BbCRASP-1 BBA68 FHBP	CRASP-2 BbCRASP-2 BBH06	CRASP-3 BbCRASP-3 BBN38	CRASP-4 BbCRASP-4 ErpC	CRASP-5 BbCRASP-5 ErpI ErpN ErpA BBP38 BBL39
Gene name		*cspA*	*cspZ*	*erpP*	*erpC*	*erpA*
Gene location in *B. burgdorferi* strain B31		lp54	lp28-3	cp32-9	cp32-2	cp32-1 cp32-5 cp32-8
Gene expression in enzootic cycle	Fed larvae	+	+ (low expression)	+ (high expression)	+ (high expression)	+ (high expression)
	Unfed nymphs	+ (high expression)	–	–	–	–
	Fed nymphs	+ (low expression)	+ (low expression)	+	+	+
	Tick biting sites	+	+ (high expression)	+ (high expression)	+ (high expression)	+ (high expression)
	Dissemination	–	+ (high expression)	+ (high expression)	+ (high expression)	+ (high expression)
FH binding	Purified proteins	+	+	+	-	+
	GOF^c^	+	+	+	–	+
	LOF^d^	+	+^e^	ND^f^	ND	ND
Additional non-FH ligands related to complement inactivation		C7, C9, FHL-1	FHL-1	CFHR1 CFHR2 CFHR5	CFHR1 CFHR2	CFHR1 CFHR2 CFHR5
Serum resistance	GOF^c^	+	+	–	–	–
	LOF^d^	+	+^e^	+^g^	–	+^g^
Infection phenotype	Spirochetes transmission by ticks	Mutant showed defects in surviving at fed nymphs and transmission to hosts	ND	ND	ND	ND
	Spirochete acquisition by ticks	–	–	ND	ND	ND
	Intradermal inoculation	–	Mutant showed defects in bloodstream survival and tissue colonization^c^	ND	ND	Mutant showed defects in tissue colonization^h^

a *Table adapted from Kraiczy and Stevenson (2013)*.

b *Different information may be shown because of different strains used to define that information. The information here is derived from B. burgdorferi strain B31*.

c *Produced in a gain-of-function background (GOF)*.

d *Produced in a loss-of-function background (LOF)*.

e *Only in blood treated condition*.

f *Not determined*.

g *Only when ErpP and ErpA are expressed under flaB promoter in a cspA-deficient B. burgdorferi in the infectious background*.

h *Performed using a transposon-inserted erpA mutant in an infectious B. burgdorferi background*.

In this review, we summarize previous findings regarding the role of CRASPs in the pathobiology and provide mechanistic insights into transmission and dissemination of LD spirochetes in ticks and different vertebrate animals.

## CspA Facilitates Spirochete Survival in Ticks' Blood Meal and During Transmission From Ticks To Hosts

During feeding, ticks are vulnerable to the attack by complement present in the blood meal. To neutralize complement and other dangerous constituents, ticks generate a cocktail of diverse immunomodulatory proteins with immunosuppressive, anti-inflammatory, and anti-complement activity in their saliva (Tyson et al., 2007, 2008; Schuijt et al., 2008, 2011; Wagemakers et al., 2016) (see de Taeye et al., 2013 for the review). These proteins shield spirochetes from complement-mediated killing in the ticks' midgut. However, ticks devoid of any one of these anti-complement proteins can still transmit spirochetes to vertebrate animals (Schuijt et al., 2011; Wagemakers et al., 2016). Additionally, LD spirochetes survive at similar levels in the ticks feeding on wild-type or complement-deficient mice (Rathinavelu et al., 2003; Hart et al., 2018). These results suggest that spirochetes have developed additional means to evade complement when residing in fed ticks.

The *cspA* gene is located on a linear plasmid 54 (lp54) which is essential for LD spirochetes survival in the infection cycle (Purser and Norris, 2000; Table 1). This gene is uniquely expressed in spirochetes residing in ticks, suggesting that CspA plays a role during spirochetal colonization of ticks (von Lackum et al., 2005; Bykowski et al., 2007; Hart et al., 2018; Table 1). Ectopically producing CspA into a non-infectious, serum-sensitive, and *cspA*-deficient *B. burgdorferi* strain enables this strain to inactivate complement and survive when exposed to sera from various vertebrate animals *in vitro* (Kraiczy et al., 2004b; Brooks et al., 2005; Hammerschmidt et al., 2014; Muhleip et al., 2018; Table 1). Conversely, deleting *cspA* from a low passage and fully infectious *B. burgdorferi* strain results in the inability of this strain to survive in presence of serum from vertebrate animals and enhances complement activation on spirochete surface (Kenedy et al., 2009; Table 1). These results demonstrate the role of CspA in conferring spirochetal evasion from complement.

Moreover, a previous study demonstrates that CspA also confers protection when spirochetes are exposed to complement components in blood acquired during tick feeding. A recent study shows that a LD *Borrelia* strain deficient in *cspA* is eliminated in nymphs after the nymphs feed on wild-type mice (Hart et al., 2018). However, this strain survives in the nymphs feeding on complement deficient mice, indicating that CspA promotes spirochetal evasion of complement in ticks' blood meal (Hart et al., 2018). The CspA-mediated blood meal survival has been attributed to the ability of CspA to bind FH (Hart et al., 2018; Figure 1 and Table 1). CspA orthologs from different LD species differ in their ability to bind to FH from other vertebrate animals including birds, mice, and humans (Bhide et al., 2009; Hart et al., 2018; Muhleip et al., 2018). CspA of *B. burgdorferi* displays <50% of sequence identity compared to other LD *Borrelia* species but >95% identity on the intra-species level (von Lackum et al., 2005; Wywial et al., 2009). Further, the sequence variability of CspA orthologs correlates with their ability to interact with FH from humans and other hosts (von Lackum et al., 2005; Bhide et al., 2009; Hammerschmidt et al., 2014; Hart et al., 2018; Muhleip et al., 2018). Of note, one previous study showed that recombinant CspA from *B. burgdorferi* B31 does not bind to non-human FH in the sera applied on a Far-Western blot (McDowell et al., 2006). This result suggests that those non-human FH variants are required to be maintained as a native form in order to display their ability to bind to CspA. Consistent with the allelic differences in FH-binding activity of CspA, a *cspA-*deficient *B. burgdorferi* strain producing CspA from *B. garinii* was incapable of surviving in nymphs upon feeding on wild-type mice (Hart et al., 2018). That isogenic strains survived in nymphs feeding on the complement-deficient mice, similar to the isogenic strain producing CspA from *B. burgdorferi* strain B31 (Hart et al., 2018). These findings imply an allelic variation of CspA-mediated FH-binding activity. Such results also lead to an intriguing possibility that CspA determines spirochete host tropism by driving the transmission from ticks to specific hosts (Kurtenbach et al., 2002; Kraiczy, 2016; Tufts et al., 2019).

Recent investigations also revealed that CspA acts in multiple ways to inactivate complement. CspA was shown to inactivate the AP by binding to FH and FHL-1 as well as by binding to complement proteins C7 and C9 to block MAC formation (Hallstrom et al., 2013; Table 1). The presence of CspA on the bacterial surface prevents the formation of MAC, suggesting a FH-independent mechanism to confer complement evasion. However, compared to the high affinity binding to FH (K_D_ < 100 nM), CspA binds only moderately to C7 and C9 (K_D_ > 5 μM). These results raise questions regarding the physiological relevance of CspA-mediated C7- and C9-binding activity (Kraiczy et al., 2004a; Hallstrom et al., 2013; Hart et al., 2018).

## The Role of CspZ in Promoting Spirochete Dissemination After Invading Vertebrate Hosts

A previous finding indicates that a *B. burgdorferi* strain deficient in *cspA* is capable of surviving at the inoculation site in skin at similar levels to the wild-type parental strain introduced by needle infection (Hart et al., 2018). This suggests that additional proteins confer this phenotype and/or work collaboratively with CspA to facilitate the establishment of infection. In fact, CspZ has been identified as an additional FH/FHL-1-binding protein which is encoded on the linear plasmid 28-3 (lp28-3) of *B. burgdorferi* B31 (Table 1). During tick-to-host transmission, the expression of *cspZ* is undetectable when spirochetes reside in ticks, but up-regulated when spirochetes reach the bite site in host skin (Bykowski et al., 2007). Further investigation reveals that *cspZ* is expressed throughout different infection stages in vertebrate animals (Bykowski et al., 2007; Marcinkiewicz et al., 2019), suggesting that the expression of CspZ and its role in the infection are restricted to the host (Table 1). Similar to CspA, introduction of CspZ into a *cspZ*-deficient, serum-sensitive borrelial strain allows the transformed strains to survive *in vitro* in presence of serum from various vertebrate animals by preventing complement activation (Hartmann et al., 2006; Siegel et al., 2008; Table 1). However, a *cspZ*-deficient strain in the infectious background of *B. burgdorferi* also survived in sera and colonized mouse tissues at similar levels as the parental strain (Coleman et al., 2008; Marcinkiewicz et al., 2019; Table 1). These findings support the following notions that such indistinguishable phenotypes could be attributed to low expression levels of *cspZ* in *B. burgdorferi* (Bykowski et al., 2007; Rogers and Marconi, 2007; Marcinkiewicz et al., 2019). As LD spirochetes produce additional complement interacting proteins that confer evasion during dissemination, delineating CspZ's phenotype can be cumbersome (Kraiczy et al., 2003, 2004a; Alitalo et al., 2004, 2005; Pietikainen et al., 2010; Bhattacharjee et al., 2013; Garcia et al., 2016; Caine et al., 2017).

To amplify the phenotype conferred by these genes, vertebrate blood has been used to cultivate spirochetes as cue to mimic *in vivo* conditions, possibly due to host-specific nutrients and ions in blood (Tokarz et al., 2004). Several borrelial genes upregulated during transmission can be triggered *in vitro* by incubation of the spirochetes with host blood (Tokarz et al., 2004). These genes include *cspZ*. These findings are consistent with additional data showing that a *cspZ*-deficient strain in an infectious background of *B. burgdorferi* displays reduced ability to survive when incubated with vertebrate sera (Marcinkiewicz et al., 2019; Table 1). Furthermore, this *cspZ* mutant strain when pre-treated with blood shows a delayed onset of dissemination and lower burdens in distal tissues, compared to wild-type *B. burgdorferi* strain, demonstrating CspZ' role in promoting spirochete dissemination (Marcinkiewicz et al., 2019; Figure 1 and Table 1).

Further, several studies examined the role of CspZ (or the plasmid encoding *cspZ*) in infection cycle. CspZ was shown not essential for spirochetes acquisition from mammalian hosts to ticks (Coleman et al., 2008). However, fewer mice develop antibody reactivity against whole spirochete cell lysates after being fed on by the ticks carrying a *B. burgdorferi* strain missing lp28-3 plasmid which encodes *cspZ*, compared to wild-type parental spirochete strain (Dulebohn et al., 2013). These findings suggest that the proteins encoded by lp28-3 (e.g., CspZ) facilitate spirochete to establish an infection and disseminate to distal sites after tick bites. A previous study revealed that LD patients with manifestations (e.g., acrodermatitis, neuroborreliosis, erythema migran) and/or positivity in two-tier LD serological tests elicited antibodies to CspZ, indicating that spirochetes produce this protein during the infection process (Kraiczy et al., 2008; Rogers et al., 2009).

Rogers et al. observed that CspZ shows allelically different ability in binding to human FH (Rogers and Marconi, 2007; Rogers et al., 2009). As CspZ is highly conserved (nearly 98% identical among *B. burgdorferi* strains and ~70% identical among LD spirochetes), the difference of these variants may convey the observed strain-to-strain variation in binding activity to human FH (Rogers et al., 2009; Brangulis et al., 2014). Several sequence diverse regions in CspZ have been identified (Brangulis et al., 2014). According to a recently reported high-resolution co-crystal structure of CspZ-FH binding complex (Protein Data Bank #6ATG) some of these variable regions are located in the binding site/interface with human FH. These results support the possibility that these variable regions of CspZ mediate the different levels of FH-binding activity and spirochete survival in the infection cycle (Table 1).

## The Role of OspE Paralogs in Spirochete Survival During the Infection Cycle Remains Unclear

Not every spirochete strain isolated from ticks feeding on LD spirochetes-infected vertebrate hosts encodes CspZ (Rogers and Marconi, 2007; Kraiczy et al., 2008), supporting that additional FH-binding proteins confer dissemination during infection. In fact, LD spirochetes produce multiple copies of OspE proteins, encoded by several circular plasmids 32 (cp32) (Marconi et al., 1996; Stevenson et al., 1996; Akins et al., 1999; Caimano et al., 2000; Kraiczy and Stevenson, 2013; Table 1). Most of these OspE paralogs bind to FH *in vitro* and share similar promoter sequences (as known as upstream homology box or “UHB”) to other outer surface proteins on cp32, such as OspF (Marconi et al., 1996; Akins et al., 1999; Caimano et al., 2000; Brissette et al., 2008). Because of these similarities, these Osp*E/*F-related proteins were grouped under the term as Erps (Brissette et al., 2008).

Although some Erps have been shown to bind FH and confer complement evasion, their role in spirochete survival during the infection remains less clear. A serum-sensitive *B. burgdorferi* strain which expresses *erpP* or *erpA* (the genes encoding OspE paralogs in *B. burgdorferi* B31) driven by the endogenous promoters, remains susceptible to complement-mediated killing in human serum (Siegel et al., 2010; Hammerschmidt et al., 2012; Table 1). This result is consistent with other *B. burgdorferi* strains (i.e., the *cspA*-deficient strain) encoding *erpP* and *erpA* under the control by the endogenous promoters which remain susceptible to human serum. However, when those genes are expressed ectopically in a serum-sensitive *B. burgdorferi* strain using a strong and constitutive promoter, these spirochetes inactivate complement and survive when incubated with human serum (Kenedy and Akins, 2011; Table 1). These results imply that high expression levels of OspE are needed for complement inactivation and serum resistance.

The genes encoding OspE paralogs are not expressed when spirochetes are in post-molting flat nymphs whereas they are upregulated immediately after blood meals (Hefty et al., 2001; Miller et al., 2003). Additionally, the expression of *ospE* is maintained throughout different stages of infection after spirochete transmission from ticks to hosts (Hefty et al., 2001; Miller et al., 2003, 2005; Table 1). Consistent with the expression profiles of these *ospE* genes, spirochete burdens are reduced in nymphs feeding on mice passively immunized with anti-OspE IgG, but remain unaffected when feeding on mice inoculated with Ig isotype control (Nguyen et al., 1994). Further, the transposon-inserted *erpA* mutant in an infectious *B. burgdorferi* strain causes a 2-week delay in dissemination to distal tissues when co-infected with a library of other transposon-inserted mutants (Lin et al., 2012; Table 1). These findings suggest that OspE paralogs may play a role in conferring tick-to-host transmission of spirochetes as well as facilitating rapid dissemination to distal tissues (Figure 1). However, the off-target silencing by antibody-dependent deletion or transposon insertion methodologies may be the confounding effects of these results. Generating the deletion mutant of *ospE* paralogs could be the favorable approach to address this caveat, but multiple copies of OspE present in LD spirochetes could be cumbersome. Thus, the gain-of-function approach such as producing these OspE paralogs in a serum-sensitive strain and evaluating bloodstream survival during a short-term infection may be a suitable approach to address these technical hurdles (Caine and Coburn, 2015).

OspE paralogs among different strains have highly variable sequences (Marconi et al., 1996; Sung et al., 1998; Akins et al., 1999; Caimano et al., 2000; Stevenson and Miller, 2003; Brissette et al., 2008). These variants differ in their ability to bind to vertebrate animals' FH (Stevenson et al., 2002; McDowell et al., 2003; Hovis et al., 2006). These results imply potential roles of OspE paralogs in promoting LD spirochetes complement evasion in a host-specific manner. Besides FH, OspE also binds to different isotypes of CFHR (Zipfel et al., 2002; Siegel et al., 2010; Kraiczy and Stevenson, 2013; Skerka et al., 2013; Jozsi et al., 2015). However, the physiological importance of CFHR-binding activity of OspE proteins is unclear and warrants further investigation.

## Conclusion

To survive their complex life cycle, LD spirochetes have developed several strategies to evade the host immune system that they encounter in ticks during feeding (blood meal) and in the bloodstream of vertebrate animals. A key evasion mechanism is to circumvent the complement components by producing complement- or CRP-binding proteins, including CRASPs, which facilitate complement inactivation. These CRASPs have been shown to confer spirochete transmission from ticks to hosts and promote infection and dissemination in vertebrate hosts. However, the concurrent production of CRASPs increases the complexity in delineating the contribution of these proteins individually in each of the stages within the infection cycle. Elucidating such mechanisms will provide new insights into how spirochetes survive in two distinct environments, ticks, and vertebrate hosts. Such information will provide foundation for the development of preventions through targeting CRASPs to block these infection mechanisms, which will ultimately reduce LD burdens in humans.

## Author Contributions

Y-PL, AF, TN, and PK wrote the manuscript. TN and Y-PL prepared the figures.

### Conflict of Interest

The authors declare that the research was conducted in the absence of any commercial or financial relationships that could be construed as a potential conflict of interest.

## Abbreviations

CRASPs: Complement regulator acquiring surface proteins
OspE: OspE paralogs
CP: Classical Pathway
LP: Mannose-binding lectin pathway
AP: Alternative pathway
TP: Terminal pathway
MAC: Membrane attacking complex
CRPs: Complement regulatory proteins
FH: Factor H
BbCRASPs: *Borrelia burgdorferi* sensu lato complement regulator acquiring surface proteins
FHL-1: Factor H like protein 1
CFHR: Factor H related protein
lp54: Linear plasmid 54
lp28-3: Linear plasmid 28-3
cp32: Circular plasmid 32
UHB: Upstream homology box
LD: Lyme diseases.
